# Motivation behind running among older adult runners

**DOI:** 10.1186/s13102-021-00366-1

**Published:** 2021-10-29

**Authors:** Patxi León-Guereño, Héctor Galindo-Domínguez, Eneko Balerdi-Eizmendi, Mateusz Rozmiarek, Ewa Malchrowicz-Mośko

**Affiliations:** 1 Faculty of Education and Sports, University of Deusto, Camino Mundaiz 50, 20012 San Sebastián, Basque Country Spain; 2 Health, Physical Activity and Sports Science Laboratory (HealthPASS), Department of Physical Activity and Sports, Faculty of Education and Sports, University of Deusto, 48007 Bilbao, Spain; 3 Facultad de Humanidades, Educación y Psicología, Universidad Francisco de Vitoria, Madrid, Spain; 4 Faculty of Physical Culture Sciences, Department of Sports Tourism, Poznan University of Physical Education, Krolowej Jadwigi 27/39, 61-871 Poznan, Poland

**Keywords:** Silver runners, Running, Amateur, Older adult, Motivation, Years’ experience, Participation, Age, Gender, Family life, Children

## Abstract

**Background:**

Recreational running has greatly increased over the last decade, and different research has tried to understand the motivation that leads these amateur athletes to run. However, most research has focused on adult athletes, while the motivation behind older adult athletes has been overlooked.

**Methods:**

The aim of this research was to analyse the motivational aspects of amateur runners aged over 50 years, and to consider the influence that years of practice, type of participation and some socio-demographical variables have on these older adults’ motivation behind running. 244 older adult amateur athletes in total completed an online survey with the 56 items contained in the motivation of marathoners scales (MOMS), 108 of whom were female (44%), and 136 were male runners (56%). Athletes were asked about their years’ running experience (< 1 year, 1–5 years, 6–10 years, > 10 years), their participation in running events (recreationally, half marathon, marathon, ultramarathon) and age (ranges 50–60, more than 60 years), gender (male, female), family life (whether in a relationship or not), and whether they had children (yes, no).

**Results:**

The results showed statistical differences in the nine MOMS dimensions in terms of years’ running experience and participation in different running events. Moreover, older adult runners’ gender and age subsequently showed statistical differences with five and six motivational factors respectively, while athletes that did not have children were more likely to run regarding competition and showing recognition. Family life did not show any statistical association with any of the dimensions on the scale in this population, while regression analyses showed that, mainly, years’ running experience and participation were positively predicted, together with most motivational dimensions, while having children was negatively predicted in some of them.

**Conclusions:**

This study showed that older adult runners’ reasons for participating differ from those obtained in previous studies, especially regarding training experience and participation in events. Therefore, the older adult population should be specifically addressed.

**Supplementary Information:**

The online version contains supplementary material available at 10.1186/s13102-021-00366-1.

## Background

Running has become one of the main physical activity in recent years [1, 2]. and research into this topic has increased exponentially, with much being carried out worldwide in relation to various diverse factors linked to this practice, e.g. physical health benefits [3–5], physiology and nutrition [1, 6] and psychological aspects of running [1, 7–10]. However, while much previous research has focused on athletes’ performance [5, 11–13], although we can find some previous research on master athletes [14] and elderly motivation toward physical activity [15] or sport participation [16], it can be said that little research has been undertaken to try and explain amateur runners’ psychological, physiological, and pathophysiological aspects. Furthermore, since participation in endurance events has greatly increased, the reasons why those athletes take part in different races has become one of the main research questions in literature on the subject [17]. Therefore, recent research has tended to be focused on trying to understand the motivation behind athletes in different endurance races such as the triathlon [18–23], cycling [24–27] or different distance running events [28].

Since motivation is considered to be one of the key factors in sport psychology [29, 30], it has tended to be associated with very different topics, such as runners’ injuries [31, 32], or even comparing the motivational aspects of runners from different countries. [33]. However, most of the research related to running motivation has been carried out on marathon runners, especially since Masters et al. [34] developed the motivation of marathoners scale (MOMS), an instrument that has been mostly used in this research area. Apart from marathoners’ motivation behind participating [29, 35–40], athletes’ reasons for participating have been analysed in ultramarathons [30, 41–43], half-marathons [44, 45], park runs and city trails [46], with urban runners [47, 48], and even by comparing different distances such as 5 k, half marathons, full marathons, and ultramarathoners [28, 33]. Moreover, this worldwide phenomenon, even retaining to some extent the original question as to why runners voluntary expose themselves to such strain [34], this type of research has been developed in different countries. E.g. Poland [36, 40], Greece [37], the United States [38, 39] and Spain [29]. In fact, some studies have recently compared these types of motivation behind running cross culturally, in an attempt to ascertain whether there are any differences in reasons for participating between United Kingdom and Indian runners [33].

Athletes’ motivation may vary depending on some sociodemographic variables, and in previous research, runners’ motivation has been mostly associated with gender [28, 36, 37, 40, 45, 46, 49–51] probably due to the lower participation of female athletes in different running events [37]. Overall, this research shows that male participants’ motivation was more likely to be linked to achievement dimensions such as competition or personal goal achievement, while women athletes’ motivation was more closely linked to social motives like affiliation or psychological motives such as psychological coping, self-esteem or the meaning of life. Athletes’ age has also been widely associated with reasons for participating on the part of recreational runners [36–38, 40, 46, 52]. Previous data has shown that the age range of runners’ is of importance when it comes to participation motives, and especially with regard to achievement motives, such as competition and personal goal achievement linked to younger athletes. On the other hand, female athletes did not evidence such differences [37]. Apart from gender and age, other sociodemographic variables such as number of children [29, 46] or athletes’ family life or marital status have been recently analysed [43, 46], with recognition motives being greater in athletes without children, and results linked to being in a relationship, affiliation and personal goal achievement. Amateur years’ running experience has also been associated with runners’ motivation [30, 40, 53, 54], since it is a variable that might influence it. In the same way, reasons for participating in different running events according to running distance have also been researched [28–33], because the motivation behind running 5 k is not necessarily the same as for running longer distances [55] such as half, full or ultramarathons.

Despite the different variables that have been used to understand amateur athletes’ reasons for participating in different running events, there has been little research into trying to identify amateur athletes’ motivation in other age ranges [56], although there may be differences due to different life stages of the participant. Even though we can find research related to master athletes’ motivation in different international sports competitions [57, 58], there is a lack of research regarding recreational older adult runners. Ogles et al. [38] compared older vs. younger adult male runners` participative motives twenty years ago, in a different social context, although in that case, female athletes over 50 years old were basically inexistent non-existent, for example. However, the number of older adult athletes is increasing across western countries [59], and despite the fact that physical activity can have a positive influence on older adults aged 50 years and older, and their health [16], their reasons for participating in these different running events remain unclear. This lack of information does not allow event organizers, personal trainers or coaches to promote the participation of this growing population.

Therefore, since older adults’ reasons for running have not been yet suitably addressed, the aim of our study was to examine amateur older adult runners’ reasons for participation, together with the association of variables such as athletes’ gender, age, number of children, family life, participation in different running events, and their years’ running experience with older adult runners’ motivation behind running.

## Methods

### Participants and study design

244 individuals in total participated in this descriptive, quantitative, cross-sectional study. Regarding personal characteristics, 108 were women and 136 men, while of the total, 182 were between 50 and 60 years old, and 62 were over 60 years old, so all participants were of age. The questionnaire was sent to runners’ clubs and seniors’ clubs from the Greater Poland region. Organized running clubs exist only in Poznan—the capital of the Greater Poland region. All the questionnaires were delivered to the respondents via the clubs (Additional file 1) (Table 1).

**Table 1:** Number of participants according to family context, type of participation and years of running experience

Family context			Participation			Years of running experience			
Sing/Div/Wid	Part/Marri	Recreation	Half marathon	Marathon	Ultramarathon	< 1 year	1–5 years	6–10 years	> 10 years
86	158	63	113	54	12	33	112	86	13

### Measurements

Following previous research on athletes’ motivation [36, 43, 46], participants were asked about the following sociodemographic variables such as, gender (male, female), age (50–60, > 60) family life (Singel/divorced/Widowed, partner/marriage) and whether they had children (no, yes).

Moreover, athletes were asked about their years’ running experience (less than 1 year, between 1–5 years, 6–10 years, more than 10 years) and the type of running events in which they take part (running recreationally or less than half marathon), half marathon, marathon, ultramarathon).

An adapted version of The motivation of marathoners scales (MOMS) was used [34]. This survey was translated into the Polish language and adapted by Dybała [60], and retained the structure of the original scale, with 9 dimensions, all of them divided into four main motivational groups: Psychological motives (meaning of life, self-esteem), achievement motives (competition, personal goal achievement), social motives (affiliation, recognition) and physical health-related motives (general health orientation, weight concern). The questionnaire included 56 items, with a 7-point Likert scale, with the highest scores or reasons for participating being 7 (7 = *most important reason*) and the lowest scores 1 (1 = *not a reason*). The original MOMS questionnaire was published in the *Research Quarterly for Exercise and Sport* in 1993 by Masters et al. [34], and its Polish version was officially released and published in 2013 by Dybała [60].

The instrument had a good model fit (X^2^/df = 2.1; CFI = 0.820; RMSEA 0.069; AIC: 2811.00), considering the complexity of the model and the quantity of items, aspects that significantly penalty the model fit [61]

### Procedure

Runners were asked via an online survey package [62, 63], being asked initially about some sociodemographic questions and followed by the 56 items from the MOMS. The questionnaire was sent to running clubs and senior clubs from the Greater Poland region, with the different clubs being suitably informed about this study beforehand. The Google Docs questionnaire survey was open for one month, from October 15, 2020 to November 15, 2020. The study was in accordance with the Helsinki declaration of 1975, and participants were treated ethically according to the American Psychological Association ethics code [64]. The study does not require formal ethical approval, because in accordance with the rules in force in Poland, the Bioethics Committee does not submit applications for surveys consisting in the use of standardized surveys, used in accordance with their intended purpose, when the research will develop statistically selected elements of the survey [65]. Our questionnaire did not require the completion of a separate participant information sheet or consent form but clearly indicated in the headline that all questionnaire takers give informed consent to the study. Respondents were informed about the course and character of the survey. The survey was anonymous, voluntary and confidential, which was enough, since in Poland, anonymous diagnostic surveys do not require approval by a bioethics committee [46].

### Data analysis

After collecting all the information and creating a database in Excel, this information was then exported to SPSS Statistics 23. At first, homogeneity through Levene’s test and normality through skewness and kurtosis was tested. In all cases, Levene’s test, for each dimension of MOMS scale was statistically non-significant, assuming homogeneity of variances. With regard to normality, the dimensions of MOMS’ skewness and kurtosis ranged from − 2 to + 2, considering acceptable values for assuming the normality of the sample [66] and accepting the usage of parametric tests.

The main analyses began with the means, standard deviations, correlations through Pearson's r statistic and reliability indices were studied for all the dimensions using Cronbach's alpha statistic. After t-tests were performed (power, *p* values and cohen's d were calculated); then a series of ANOVAs were performed (power, *p* values, Tukey post-hoc tests, and cohen's d were calculated); and finally in order to ascertain the influence of personal, contextual, family and sports variables on the main dimensions of the MOMS instrument, a series of regression analyses were carried out (indicating the coefficients of determination R^2^; power; unstandardized beta values and *p* values), with all these variables being understood as independent variables, and the different dimensions of the MOMS instrument as dependent variables.

## Results

### Older adults’ motivation to run

Table 2 shows the mean scores and standard deviation of the nine dimensions of the MOMS and the correlation among those motivational dimensions. The greatest motivation behind running in older adult amateur runners are connected to physical health dimensions; general health orientation (M = 4.84, SD ± 0.612) and weight concern (M = 4.74, SD ± 0.924), and to psychological motives such as self-esteem (M = 4.80, SD ± 0.631) and psychological coping (M = 4.64, SD ± 0.596), while the lowest scores regarding reasons for participating are related to social motives such as affiliation (M = 4.00, SD ± 1.16) and achievement motives such as competition (M = 4.14, SD ± 0.1.32).

**Table 2:** Mean scores, standard deviation, and correlations of the nine dimensions of the MOMS

	M	DT	1	2	3	4	5	6	7	8	9
Self-esteem	4.80	0.631	(0.816)	0.798**	0.812**	0.741**	0.670**	0.724**	0.634**	0.533**	0.129*
Psychological coping	4.64	0.596		(0.835)	0.798**	0.609**	0.545**	0.639**	0.516**	0.556**	0.330**
Meaning of life	4.51	0.678			(0.840)	0.707**	0.675**	0.736**	0.622**	0.500**	0.210**
Personal goal achievement	4.59	0.901				(0.860)	0.825**	0.795**	0.695**	0.327**	− 0.089
Competition	4.14	1.32					(0.894)	0.853**	0.835**	0.304**	− 0.182**
Showing recognition	4.24	1.09						(0.901)	0.811**	0.258**	− 0.033
Affiliation motives	4.00	1.16							(0.918)	0.406**	− 0.116
General health orientation	4.84	0.612								(0.728)	0.292**
Weight concern	4.74	0.924									(0.785)

*p < 0.05; **p < 0.01

### Years of running experience and type of participation

Table 3 shows that older adult athletes’ years’ running experience is statistically significant in the nine dimensions of the MOMS (*p* < 0.05). Likewise, participation in different running events shows statistical differences in the nine dimensions on the scale (*p* < 0.01).

**Table 3:** Group differences

	Years of running				Participation			
	*p*	η^2^	1–β	Post-Hoc	*p*	η^2^	1–β	Post-Hoc
Self-esteem	< 0.001	0.237	1.00	(0 < 1)(0 < 2)(0 < 3)	< 0.001	0.339	1.00	(0 < 1)(0 < 2)(0 < 3)
Psychological coping	< 0.001	0.118	0.999	(0 < 1)(0 < 2)(0 < 3)	< 0.001	0.187	1.00	(0 < 1)(0 < 2)(3 < 1)(3 < 2)
Meaning of life	< 0.001	0.171	1.00	(0 < 1)(0 < 2)(0 < 3)	< 0.001	0.281	1.00	(0 < 1)(0 < 2)(3 < 1)(3 < 2)
Personal goal achievement	< 0.001	0.286	1.00	(0 < 1)(0 < 2)(0 < 3)(1 < 2)	< 0.001	0.446	1.00	(0 < 1)(0 < 2)(0 < 3)
Competition	< 0.001	0.328	1.00	(0 < 1)(0 < 2)(0 < 3)(1 < 2)	< 0.001	0.645	1.00	(0 < 1)(0 < 2)(0 < 3)(3 < 1)(3 < 2)
Showing recognition	< 0.001	0.269	1.00	(0 < 1)(0 < 2)(0 < 3)(1 < 2)	< 0.001	0.569	1.00	(0 < 1)(0 < 2)(0 < 3)(3 < 1)(3 < 2)
Affiliation motives	< 0.001	0.358	1.00	(0 < 1)(0 < 2)(0 < 3)(1 < 2)	< 0.001	0.625	1.00	(0 < 1)(0 < 2)(0 < 3)
General health orientation	< 0.001	0.110	0.998	(0 < 1)(0 < 2)(0 < 3)	< 0.001	0.098	0.994	(0 < 1)(0 < 2)
Weight concern	0.004	0.054	0.885	(0 < 1)(0 < 2)	< 0.001	0.221	1.00	(1 < 0)(2 < 0)(3 < 0)(1 < 3)(2 < 3)

One-way ANOVA of the Motivation of Marathoners Scale’ nine dimensions (MOMS) and ranges of years’ running experience, and type of running events

Post-Hoc with Tukey Post-Hoc analysis. Years’ running: (0) Less than 1 Year, (1) 1–5 Years, (2) 6–10 Years, (3) More than 10 years; Participation in running events: (0) Running for recreation, (1) Running a half marathon, (2) Running a marathon, (3) Running an ultra-marathon

### Older adults’ motivation to run according to their age and gender

Age differences among older adult athletes show statistical differences in six out of the nine dimensions of the MOMS (Table 4): self-esteem (*p* < 0.01), personal goal achievement, (*p* < 0.05), competition (*p* < 0.01), recognition (*p* < 0.01), affiliation (*p* < 0.01), weight concern (*p* < 0.01).

**Table 4:** Group differences

	Age							Gender						
	50–60		> 60		t-test			Women		Men		t-test		
	M	SD	M	SD	1–β	*p*	d	M	SD	M	SD	1–β	*p*	d
Self-esteem	4.86	0.581	4.61	0.731	0.787	0.006	0.37	4.69	0.667	4.89	0.588	0.689	0.014	0.31
Psychological coping	4.65	0.577	4.59	0.650	0.112	0.468	0.09	4.60	0.567	4.67	0.617	0.153	0.353	0.14
Meaning of life	4.55	0.638	4.38	0.776	0.388	0.130	0.23	4.50	0.685	4.51	0.675	0.053	0.874	0.01
Personal goal achievement	4.68	0.866	4.35	0.965	0.695	0.014	0.35	4.37	1.02	4.77	0.743	0.977	0.001	0.44
Competition	4.33	1.20	3.59	1.50	0.972	0.001	0.54	3.96	1.40	4.28	1.23	0.467	0.065	0.24
Showing recognition	4.37	1.04	3.87	1.15	0.879	0.002	0.45	4.08	1.17	4.37	1.01	0.552	0.040	0.26
Affiliation motives	4.13	1.07	3.63	1.33	0.840	0.009	0.41	3.82	1.31	4.15	1.01	0.587	0.035	0.28
General health orientation	4.85	0.608	4.81	0.627	0.072	0.666	0.06	4.86	0.563	4.83	0.650	0.069	0.685	0.04
Weight concern	4.64	0.905	5.06	0.914	0.878	0.002	0.46	4.99	0.695	4.55	1.03	0.962	0.000	0.50

T-TEST comparisons among MOMS’ nine dimensions and older adult athletes’ ages, and gender

Having children or not shows statistical differences in competition motives (*p* < 0.01) and recognition motives (*p* < 0.01) in terms of participation in different running events. Table 4, shows statistical differences in five out of nine motivational dimensions of the MOMS according to the gender, personal goal achievement with higher values for men (4.77), and weight concern motives showing higher values for women (4.99) (*p* < 0.01), self-esteem, recognition and affiliation motives showed higher values for women, making it more likely that they will take part in running events (*p* < 0.05). Conversely, marital status or being in a relationship or not does not show any statistical difference regarding the reasons for participating in older adult athletes.

### Sociodemographic variables that predict MOMS dimensions

In order to process with the regression analyses their assumptions were studied: As previously mentioned, normality and homoscedasticity assumptions were ensured in the sample distribution; as well as non-multicollineality, due to low VIF values (< 10), and independence of errors, due to Durbin–Watson values between 1.5 and 2.5 [67]. In addition, linearity was ensured by testing all the different partial regression plots.

Table 5 shows how years’ running experience is a major predictor of older adult motivation behind running, showing statistical significance in all the dimensions except weight concern. This variable positively predicts older adult athletes’ motivation related to self-esteem (β = 0.196; *p* = 0.000), psychological coping (β = 0.230; *p* = 0.000), meaning of life (β = 0.212; *p* = 0.000), personal goal achievement (β = 0.296; *p* = 0.000), competition (β = 0.414; *p* = 0.000), showing recognition (β = 0.335; *p* = 0.000), affiliation (β = 0.495; *p* = 0.000) and general health orientation (β = 0.231; *p* = 0.000).

**Table 5:** Stepwise regression analyses to predict older adult motives according to demographical variables

	Assumptions		Regression		Correlation
	VIF	Durbin–Watson	β	*p*	r
*(DV: Self-esteem,* R^2^ = 0.214; 1–β = 1.00)					
Participation	1.483	1.832	0.216	0.000	0.418**
Years’ running	1.483		0.196	0.001	0.400**
*(DV: Psychological coping,* R^2^ = .089; 1–β = .998)					
Years’ running	1.000	1.644	0.230	0.000	0.298**
*(DV: Meaning of life,* R^2^ = .126; 1–β = .999)					
Years’ running	1.483	1.920	0.212	0.001	0.330**
Participation	1.483		0.128	0.035	0.293**
*(DV: Personal goal achievement,* R^2^ = .301; 1–β = 1.00)					
Participation	1.485	1.506	0.388	0.000	0.490**
Years’ running	1.484		0.296	0.000	0.446**
Children	1.005		− 0.257	0.009	− 0.106
*(DV: Competition,* R^2^ = .406; 1–β = 1.00)					
Participation	1.485	1.737	0.652	0.000	0.558**
Children	1.005		− 0.554	0.000	− 0.171**
Years’ running	1.485		0.414	0.000	0.476**
Age	1.005		− 0.354	0.000	− 0.243**
*(DV: Showing recognition,* R^2^ = .320; 1–β = 1.00)					
Participation	1.484	1.723	0.494	0.000	0.494**
Children	1.005		− 0.449	0.060	− 0.168**
Years’ running	1.485		0.335	0.000	0.435**
*(DV: Affiliation,* R^2^ = .404; 1–β = 1.00)					
Participation	1.484	1.748	0.534	0.000	0.554**
Years’ running	1.485		0.495	0.000	0.533**
Children	1.005		− 0.383	0.007	− 0.121
*(DV: General Health Orientation,* R^2^ = 0.085; 1–β = .997)					
Years’ running	1.000	2.012	0.231	0.000	0.292**
*(DV: Weight concern,* R^2^ = 0.211; 1–β = 1.00)					
Participation	1.000	1.680	− 0.51	0.000	− 0.459**

Stepwise method used only with entrance variables. Participation in running events: (0) Runnin for recreation, (1) Running a half marathon, (2) Running a marathon, (3) Running an ultra-marathon; Age: (0) 50–60 Years, (1) 60 + Years; Gender: (0) Woman, (1) Man; Years’ running: (0) Less than 1 Year, (1) 1–5 Years, (2) 6–10 Years, (3) More than 10 years; Family life: (0) Single/divorced/widowed, (1) In partnership/married; Children: (0) No, (1) Yes; DV: Dependent Variable. *p < 0.05; **p < 0.01

Participation in different types of running event enters the equation and predicts all the motivational dimensions except psychological coping and general health orientation. This variable positively predicts older adult athletes’ motivation related to self-esteem (β = 0.216; *p* = 0.000), meaning of life (β = 0.128; *p* = 0.000), personal goal achievement (β = 0.388; *p* = 0.000), competition (β = 0.652; *p* = 0.000), showing recognition (β = 0.494; *p* = 0.000) and affiliation (β = 0.534; *p* = 0.000), while this variable negatively predicts weight concern (β =  − 0.51; *p* = 0.000).

Table 5 shows that having children or not negatively predicts older adults’ motivation related to personal goal achievement (β =  − 0.257; *p* =  − 0.106), competition (β =  − 0.554; *p* = 0.000), recognition (β =  − 0.449; *p* = 0.000) and affiliation (β =  − 0.383; *p* =  − 0.121). Lastly, age negatively predicts competition reasons for running (β =  − 0.354; *p* = 0.000).

## Discussion

The reasons why amateur athletes run can be very heterogeneous, and this is why previous research has tried to describe these types of motivation. Although most of the research focused on adult athletes, amateur athletes’ motivation have not been suitably addressed in another age ranges [56]. Within this context, the aim of this study was to analyse and study more in depth the motivation behind participating on the part of older adult athletes, more and more of whom have been taking part in different running events over the last decade.

### Older adults’ motivation to run

Our findings suggest that the greatest motivation behind running among older adult athletes were physical health-oriented dimensions, namely, weight concern and general health orientation (M = 4.84, SD ± 0.612). Partially in line with our results, previous research showed that general health-oriented reasons for participating were the main reasons why adult athletes take part in different running events [29, 30, 37, 39, 40]. However, as observed in the results of the present study, older adults obtained higher scores in weight concern motives (M = 4.74, SD ± 0.924) than in any scores we were able to find in previous research [29, 37]. Moreover, our results show that personal goal achievement motives was the fifth most common reason why older adults participate, while previous research shows, that this dimension obtained the highest or the second highest score among adult runners [30, 37, 39, 40]. Older adult runners scored highly in psychological motives, and especially self-esteem (M = 4.80, SD ± 0.631), which is in line with some of the previous research carried out in the Spanish context [29] and in the Polish context [40]. On the other hand, our results showed that social motives were the least demanded by older athletes, e.g. affiliation (M = 4.00, SD ± 1.16), with these results being similar to previous research in children and adolescents [56], and to the results obtained in the original research by Master and Ogles [39]. Lastly, our results showed that recognition motives of older adult runners (M = 4.24, SD ± 1.09), are higher than in previous research, since in particular scored the lowest in adult athletes [29, 37, 40].

### Years of running experience and type of participation

As observed in the results of the present study, older athletes’ years’ running experience shows statistical differences in all the nine dimensions of the MOMS (*p* < 0.001), i.e., depending on these athletes’ running experience, the reasons why these athletes run are statistically different. These results are partially in line with Master et al. [54], who showed that veteran runners were more likely motivated by social identity, mid experienced athletes by performance aspects and rookie marathon runners were more health and weight concern orientated. However, results obtained in this research show the opposite of those obtained by Malchrowicz-Mośko et al. [53] in recent research, in that they did not find any statistical differences in any of the MOMS dimensions regarding years’ running experience in marathon runners in the Polish context. Our findings with regards to older adult runners’ motivation, according to different running distances, showed statistical differences in the nine MOMS motivation dimensions (*p* < 0.001), thus showing, that older adult athletes’ motivation varies depending on the distance they run. Along these lines, Hanson et al. [28], who compared motivation behind running on the part of half marathoners, full marathoners and ultramarathoners, found that ultra-marathoners scored lower on health orientation and weight concern and higher on meaning of life, while full marathoners scored higher on personal goal achievement, which is partially in line with our research.

### Older adults’ motivation to run according to their age and gender

The findings with regards to older adults’ motivation behind participating in terms of age and gender show the importance of these two variables, evidencing statistical significance in six of the nine motivational dimensions in the first one, and with gender showing statistical differences in five of the dimensions. Our results suggested that self-esteem (*p* < 0.05), recognition (*p* < 0.05), and affiliation (*p* < 0.05) reasons for participating showed significant differences in terms of age and gender. Likewise, personal goal achievement (*p* < 0.001), showed that motivation scores were higher for men between 50–60 years old, while and weight concern (*p* < 0.001) for elder women over 60 years old. Therefore, these results lead us to consider that these two variables may be linked to such motivational reasons on the part of older adult athletes. At the same time, competition motives (*p* < 0.001) showed differences in terms of age, being the eldest runners less competitive, while regarding gender, these differences were not found. In line with these results, in previous research with marathoners, gender showed statistical differences with regard to personal goal achievement and self-esteem [36, 37], while age-related results in our research show that six motivational dimensions are deemed to be of importance in older adults, which is partially in line with recent studies [36], in which health orientation, affiliation and self-esteem motivation-related reasons showed statistical significance. In the classic research by Ogles and Masters [38], in line with our findings, older athletes of over 50 years of age reported being more motivated by weight concern, and significant differences were found in personal goal achievement, thus supporting the results obtained in our study. These authors also found differences in affiliation motives, meaning of life and general health orientation motives, with these last two dimensions not being in line with our research. At the same time, it is noteworthy in our results that age-related motivation differences scored more highly in favour of younger athletes, and so, as age increases, the level of motivation in terms of the different significant dimensions increases in all cases, except in weight concern, which is a motivational aspect that becomes more important in older athletes over 60 years old. As far as gender is concerned, our findings suggest that there is a tendency in favour of men in all significant dimensions (*p* < 0.05), except in weight concern (*p* < 0.001), to gain a higher score than women in motivational aspects. The results obtained regarded regarding family context, a field that has not been suitably addressed yet [68], show that family life and marital status do not influence motivation behind older adult runners, i.e., being in a relationship or not does not change when it comes to motivation behind running, with these results being in line with recent research [36, 43] insofar as no differences were found according to family life among marathon and ultramarathon runners. On the other hand, and within the family context, having children or not showed statistical differences in competition (*p* < 0.05) and recognition (*p* < 0.05) reasons why older adult runners participate, with higher scores in favour of those who had no children. Partially in line with these results, Malchrowicz-Mośko and Waśkiewicz [43] did not find any difference between adult ultramarathoners who had children and those who did not, even though a level of statistical tendency was found in competition in favour of those runners who had no children, partially in line with our research. The same author, in recent research related to park runs and city trails, found that recognition motives were significant in favour of runners who had no children [46], a correlation that was also found in our results, in which older adult runners with no children were considered more motivated with regard to recognition aspects. The results obtained from the regression analyses showed that there are three main independent variables that significantly predicted motivation behind running, these being the degree of participation in running events and the amount of years spent running, which predicted the vast majority of dimensions positively, and having children, which predicted motivation behind running less frequently and negatively.

### Limitations and prospective

The study has some limitations, such as the number of participants and contextual aspects, and so caution would be needed when generalizing about these results, since more data and its collection in other countries could lead to a different result. Likewise, the cross-sectional study design was another limitation, since it does not allow causal relationships to be established between the study variables. Despite these limitations, very few studies have focused on amateur older adult athletes, which is at the same time a strong point of the study and its special feature, since older adult amateur athletes’ motivation behind running has not been suitably addressed yet in literature. In line with Malchrowicz-Mośko and Waśkiewicz [43], who found differences between single runners over 35 and under 35 years old, with affiliation being significantly greater in older runners, it would be interesting to analyse whether age differences are of importance among single athletes, and among runners in a relationship.

## Conclusion

Based on the arithmetic means of the different dimensions of the MOMS, it has been observed that older runners tend to obtain higher scores in terms of physical health and psychological aspects, while achievement and social motives are less significant in older athletes over 60 years old. At the same time, we confirmed that age and gender are to a great extent significant when it comes to reasons why older adult runners take part. Moreover, weight concern-related motivation marks a clear line in relation to age and gender, showing higher motivation by older women. For their part, years’ running experience and participation seem to be important aspects to be considered within this population, while family life and having children or not would seem to be of less importance. In summary, this study provides important information regarding motivational aspects of older adult runners, and this knowledge would be of a great interest to running event organisers and personal trainers or coaches, in order to enhance older adult runner’s motivation behind running.

## Supplementary Information

**Additional file 1.** Motivation Older adult runners. This data file shows the information collected for this research, gathering this way all the variables and information that has been analysed for this investigation.

## Data Availability

All the data that support the results can be found in the manuscript and the additional file.

## Abbreviation

MOMS: Motivation of marathoners scale
